# The Use of Tuberculin in Poor-Law Infirmaries

**Published:** 1913-03-29

**Authors:** G. J. Masson Martin

**Affiliations:** Senior Assistant Medical Officer, Portsmouth Infirmary.


### THE USE OF TUBERCULIN IN POOR-LAW INFIRMARIES.

By G. J. MASSON MARTIN, L.R.C.P. & S.I., D.P.H., Senior Assistant Medical Officer, Portsmouth Infirmary.

There can be no doubt that the greatest number of cases of active disease that call for prolonged treatment in Poor-Law infirmaries are caused, directly or indirectly, by the tubercle bacillus. The reason for this is not far to seek. It is especially amongst the poorest, the most ill-nourished, and those exposed to the most unhygienic conditions that this organism finds its most fertile breeding-ground, and it is from this class that cases in workhouse infirmaries are drawn. It therefore behoves those who have the medical charge of such patients to try by every means in their power to combat the ravages of this disease.

In former years the only weapons which we had at our command were—in the case of tuberculosis of the lungs—good food, nutrients such as cod-liver oil, and the treatment of symptoms by drugs. In very few instances was open-air accommodation provided, and in many cases—indeed, in most cases—such patients were scattered through the wards of the infirmary, and not very much attempt made to protect the other patients in the same wards from infection. In the case of other forms of tuberculosis, such as hip disease, besides the means already mentioned the only other treatment available was by immobilisation, and, in suitable cases, surgical measures.

### Twelve Months with Tuberculin.

At present, although conditions have been improved in many institutions, the opportunities for open-air treatment are still lacking in most Poor-Law infirmaries. Of late years, however, another weapon has been added to our armoury—namely, Koch's tuberculin. Whether this remedy is a trusty and efficient weapon is still open to doubt, and it is very desirable that its efficacy should be given a fair trial by those who have such an immense amount of material at their command. Tuberculin has been in use at this infirmary at Portsmouth for about the past twelve months, and the results so far go to prove that in many cases it is a very valuable adjunct in the treatment of tuberculosis. It is too early to say definitely that complete cure has been obtained in any cases, as the possibility of relapse must not be overlooked, but many cases have been relieved and apparent cure obtained where the disease had resisted other forms of treatment.

The object of this article, however, is not to laud the virtues of tuberculin, but to point out the desirability of those who have cases of tuberculosis under their care in workhouse infirmaries of testing for themselves the value of a remedy which is still on its trial in this country, when facilities for such a test are so ready to their hand.

For this purpose they possess an advantage over medical officers in sanatoria, in that the patients in infirmaries will have to depend for specific treatment on tuberculin alone for their cure, without the aid of open-air methods, which have been proved to be of such undoubted value in this disease. They also have a much better chance of judging the effects of the remedy than those who have charge of tuberculin dispensaries, in that they have the patient continuously under their eye, with proper diet and skilled nursing. Results obtained under these latter conditions must be much more satisfactory and capable of affording reliable statistics than those obtained from patients who have to live at home and take their own temperatures. Cases in infirmaries also show a greater divergence in the forms of tuberculosis which are present than those in other institutions, and consequently these are greater opportunities for trying the remedy on all descriptions of this disease than can be obtained elsewhere.

### Infirmaries as Training Grounds.

Besides the advantages which may accrue from the use of tuberculin in infirmaries from the standpoint of the welfare of the patient, there are also advantages to the medical staff which must not be overlooked. One of the functions of a modern large Poor-Law infirmary, besides the care of the sick poor, is the adequate training of probationer nurses, and also of granting experience to newly qualified medical men. This function cannot be fully carried out if all the newest forms of treatment are not practised, and the treatment of tuberculosis by tuberculin is now a recognised form of treatment, although authorities may differ as to its value. Of late years, also, an immense amount of attention—and well-merited attention—has been directed to the subject of the cure of tuberculosis, and the general public have come to look for a higher standard of efficiency in dealing with this disease from private practitioners than formerly. Large numbers of tuberculosis dispensaries also have sprung up throughout the country, and one of the qualifications for doctors and nurses in charge of these institutions is some knowledge of the use of tuberculin. This knowledge can be obtained *par excellence* by a personal practical experience of the drug, and nowhere more readily than in the wards of a modern workhouse infirmary.

There are several objections, however, to the use of this remedy in infirmaries which may arise in the minds of those unfamiliar with the treatment. One is on the score of expense. The initial outlay for starting a course on, say, a dozen patients (and this number will be quite enough until the novice has obtained some experience) need not be more than a sovereign. All that is necessary is a 10 cubic centimetre measure divided into c.c., a "Record" syringe holding 1 c.c., and divided into one-fiftieth of a c.c. (price 8s. 6d.), a set of small bottles to hold the dilutions, and a small quantity of the sort of tuberculin which it is decided to use.* None of the commoner varieties in use cost more than 1s. 6d. a c.c., and in most cases less, with the exception of T. R., and as this preparation has been shown to have no marked advantage over the cheaper kinds it need not be used. As a rule, only minute proportions of a c.c. are injected throughout the course, and hardly ever more than 1 c.c., so that it will be seen that the cost can never be said to be heavy, and even the most parsimonious Board of Guardians can hardly object to the treatment on this account.

Another objection which might be put forward is lack of skill on the part of the medical officer who has had no former experience in the method of administration of the drug. A conscientious study of any book on the subject such as that of Bandelier and Roepke† is all that is necessary, and if the methods there advocated are adhered to, no evil consequences can result. Individual discrimination, which is so necessary for successful treatment, can be obtained only by personal experience.

A third objection which might be advocated is that cases which occur in workhouse infirmaries of tuberculosis, especially of tuberculosis of the lungs, are too advanced for treatment by tuberculin, and that mixed infection is in nearly all cases present. This is in some cases true, but very many cases present themselves in these days of improved diagnosis which are in the first and second stage, and even cases in the third stage of phthisis, which seem hopeless, can sometimes be benefited by tuberculin. In other forms of tuberculosis the drug seems to be of benefit, no matter what stage of the ailment is present. It may also protect the patient from subsequent attacks of disease after surgical measures have been taken.

Mention must also be made of the use of tuberculin in diagnosis. It has been proved beyond doubt to be of service in this respect, but most of the cases which are found in workhouse infirmaries are only too surely tubercular, and need no delicate test to demonstrate their nature.

Whatever opinion a medical officer of an infirmary who has not tried tuberculin for himself has formed of its efficacy from the writings and experience of others, he cannot deny that there is a great field of material ready to his hand on which to exploit the remedy, and the aim and hope of curing even a few cases under his care ought to be sufficient to cause him to try to aid in the amelioration and possible cure of this dread disease, which is so widespread, and the treatment of which forms so large a part of his daily work.

* Tuberculin is supplied by Meister, Lucius and Brüning, Ltd., 3 Jewry Street, E.C. A very useful pamphlet is supplied also by this firm, which gives very valuable information as to the preparation and method of administration of the different varieties of tuberculin.

† *Tuberculin in Diagnosis and Treatment*, by Bandelier and Roepke. Translated by E. C. Morland, M.B., etc. John Bale, Sons, and Danielsson, Ltd., 83-91 Great Titchfield Street, Oxford Street, W. Price 7s. 6d.

